# An Efficient and Scalable Algorithm to Mine Functional Dependencies from Distributed Big Data

**DOI:** 10.3390/s22103856

**Published:** 2022-05-19

**Authors:** Wanqing Wu, Wenyu Mao

**Affiliations:** 1 College of Cyber Security and Computer, Hebei University, Baoding 071000, China; wuwanqing8888@126.com; 2 Key Laboratory of High Trusted Information System in Hebei Province (Hebei University), Baoding 071000, China

**Keywords:** data mining, functional dependency, distributed computing, big data

## Abstract

A crucial step in improving data quality is to discover semantic relationships between data. Functional dependencies are rules that describe semantic relationships between data in relational databases and have been applied to improve data quality recently. However, traditional functional discovery algorithms applied to distributed data may lead to errors and the inability to scale to large-scale data. To solve the above problems, we propose a novel distributed functional dependency discovery algorithm based on Apache Spark, which can effectively discover functional dependencies in large-scale data. The basic idea is to use data redistribution to discover functional dependencies in parallel on multiple nodes. In this algorithm, we take a sampling approach to quickly remove invalid functional dependencies and propose a greedy-based task assignment strategy to balance the load. In addition, the prefix tree is used to store intermediate computation results during the validation process to avoid repeated computation of equivalence classes. Experimental results on real and synthetic datasets show that the proposed algorithm in this paper is more efficient than existing methods while ensuring accuracy.

## 1. Introduction

In the information age, data has become the most important asset of a company, and data-driven decisions can bring good results to every organization and company [1]. However, with the explosive growth of data volume and the variety of data sources, low quality data inevitably appears. Specifically, the collected data may contain missing, redundant, and semantic contradictions. For example, in the process of interaction with the environment, the sensor network is easily damaged under the influence of the natural environment such as sunlight and rain, resulting in equipment failure and an inability to return data or leading to the return of incorrect data [2]. Business decisions made with low-quality data can lead to huge financial losses and irreversible consequences [3,4].

Therefore, data cleaning has grown up to be a necessary prerequisite for designing and completing system engineering and has received extensive attention from many scientific researchers and related practitioners. According to statistics, in applications such as machine learning and data mining, researchers spend more than 60% of their time and energy on data preprocessing [5]. It can be seen that the theory and method of improving data quality have significant research significance and value.

Taking measures at the data source to avoid the generation of low-quality data is usually not achievable, so the main method to improve data quality is to perform error detection and repair on the dataset [6]. Many scholars have studied the process of automatic data detection and repair, including outlier detection [7,8,9], dependency conflict detection [10,11,12], and duplicate value detection [13,14]. Dependency-based methods [10,11,12] detect errors and repair data through semantic relations between data, which are represented by various integrity constraints [15], such as functional dependencies [16], conditional functional dependencies [17], and denial constraints [18]. Detecting and repairing data through dependency-based methods [19,20] still requires end-user input of integrity constraints, and the system utilizes these inputs to detect units that conflict with dependencies. However, manually writing integrity constraints are inefficient and requires sufficient domain knowledge, so it is usually necessary to mine the dependencies on the dataset with the help of automatic discovery algorithms.

Functional dependency [15] is one of the most basic and important integrity constraints. In the process of using functional dependencies to improve data quality, the primary problem is how to efficiently and automatically discover functional dependencies from table data. The study in [21] shows that the existing seven important functional dependency discovery algorithms are only suitable for small-scale centralized data sets, and cannot be extended to table data with hundreds of columns or millions of rows, and in the case of data distributed storage, these algorithms can lead to erroneous results. Therefore, with the advent of the era of big data, the amount of data has increased dramatically, and the wide application of distributed databases has brought new problems and challenges to functional dependency discovery.

**Figure 1:**
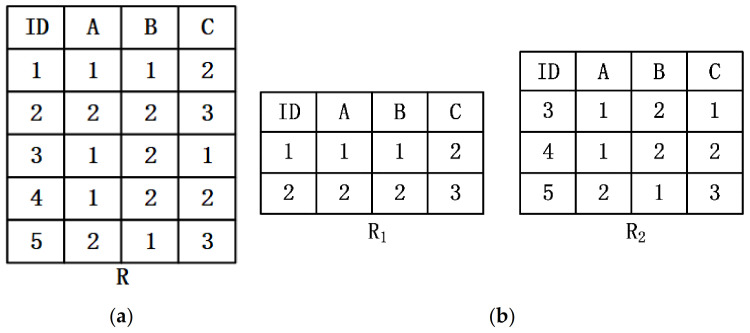
(**a**) Relation R; (**b**) The horizontal segmentation $R_{1}$ and $R_{2}$ of R.

**Example** **1.**  *Given a relation R, as shown in Figure 1a, R is horizontally divided into two parts $R_{1}$ and $R_{2}$ and distributed on different nodes, as showen in Figure 1b. According to Figure 1b, for any two tuples $t_{i}$ and $t_{j}$ on $R_{1}$ or $R_{2}$, $t_{i}\left[A\right]=t_{j}\left[A\right]$, then $t_{i}\left[B\right]=t_{j}\left[B\right]$, the functional dependency A→B can be obtained holds on either $R_{1}$ or $R_{2}$.*

However, functional dependencies that pass local validation do not necessarily hold on global data. According to Figure 1a, it can be concluded that the functional dependence A→B does not hold on R. Therefore, the existing centralized functional dependency discovery algorithms cannot be directly applied to the distributed environment.

In distributed scenarios, functional dependency discovery for large-scale data has gradually become a research hotspot. In the distributed functional dependency discovery algorithm, FDcent_discover [22] presents a distributed database functional dependency discovery framework. Firstly, functional dependency discovery is performed at each node, and then the data of each node is sent to the master node, where the centralized discovery algorithm is used to discover. The HFDD [23] and FDPar_Discover [24] algorithms adopt the data redistribution scheme to group candidate functional dependencies using the left-hand features of the functional dependencies, and send the tuples with the same common attribute values to the same node, the functional dependency discovery algorithm is performed in parallel at each node. However, there are still the following problems: First, the characteristics of the data set itself are not considered, which leads to the verification of many invalid function dependencies and increases the computational cost. Second, in distributed scenarios, when the distribution of attribute values is uneven, the load unbalance is expected to result in a waste of computing resources. Third, the repeated computation of equivalence classes in the process of verifying candidate functional dependencies leads to inefficiency.

The contributions of this paper are as follows:

1. A spark-based distributed functional dependency discovery algorithm is proposed.
2. Aiming at the unbalanced load caused by the uneven distribution of attribute values, the greedy-based task assignment strategy is proposed to balance the computing tasks of each node and avoid the unbalanced load causing too long computing time.
3. A dynamic memory management strategy is proposed to store calculated equivalence classes in memory and periodically clear equivalence classes that have not been accessed for a long time to maximize the use of memory space.
4. Verify the distributed functional dependency discovery algorithm proposed in this paper on real and artificial data sets through experiments.

This paper is organized as follows. In Section 2, the definitions and related work are introduced. Section 3 presents the algorithm structure and implementation process of the algorithm DisTFD. Section 4 presents the experimental results and the comparison of existing methods. Section 5 is the conclusion and outlook for future work.

## 2. Preliminaries

### 2.1. Definition

This section introduces definitions related to FD discovery. Let R be a relational schema and r be an instance on R. $t\left[X\right]$ represents the projection of a tuple t in R onto the subset X⊆R.

**Definition 1.**  *Functional dependency. A functional dependency X→A specifies that the value of X functionally determines the value of A, where X⊆R and A∈R. If all tuple pairs $t_{1},t_{2}\in r$ in R satisfy $t_{1}\left[X\right]=t_{2}\left[X\right]$, then $t_{1}\left[A\right]=t_{2}\left[A\right]$, then the functional dependency X→A on the instance r of R is established. Let X be the left part (LHS) of the FD and A be the right part (RHS) of the FD.*

**Definition 2.**  *Non-trivial functional dependency. If a functional dependency X→A holds and A∉X, then X→A is said to be a non-trivial functional dependency.*

**Definition 3.**  *Minimum functional dependency. If a functional dependency X→A holds and any proper subset $\bar{X}$ of X cannot determine the value of attribute A, that is, for any $\bar{X\;}\in X$, $\bar{X\;}\in X\in A$ does not hold, then we call X→A the minimum functional dependency.*

**Definition 4.**  *Equivalence class. The equivalence class of a tuple t∈r is expressed as ${\left[t\right]}_{X}=\{u\in r|\forall A\in X\;t\left[A\right]=u\left[A\right]\}$. Taking the relation R in Example 1 as an example, an equivalence class of the tuple $t_{1}$ on the attribute C is $\left\{1,4\right\}$.*

**Definition 5.**  *Partition. Divide all tuples in r into multiple equivalence classes based on the attribute set X∈R. The partition $\Pi_{X}=\{{\left[t\right]}_{X}|t\in r\}$ of relation r on attribute set X is the set of all equivalence classes, and $\left|\Pi_{X}\right|$ represents the number of equivalence classes in $\Pi_{X}$. In Example 1, the relation R can be divided into multiple equivalence classes on the attribute set $\left\{C\right\}$: $\Pi_{C}=\left\{\left\{1,4\right\},\left\{2,5\right\},\left\{3\right\}\right\}$, $\left|\Pi_{C}\right|=3$.*

**Definition 6.**  *Stripped partition. The stripped partition $\hat{\Pi_{X}}$ of relation r on attribute set X refers to the partition obtained by removing all equivalence classes with 1 element on the basis of $\Pi_{X}$. In Example 1, the relation R is divided into $\Pi_{C}=\left\{\left\{1,4\right\},\left\{2,5\right\},\left\{3\right\}\right\}$ based on the attribute set $\left\{C\right\}$, then its stripped partition $\hat{\Pi_{C}}=\left\{\left\{1,4\right\},\left\{2,5\right\}\right\}$.*

### 2.2. Related Work

Functional dependency discovery. Existing functional dependency discovery algorithms are mainly used in centralized environments and can be divided into three categories: lattice search algorithms, difference and consensus set algorithms, and hybrid algorithms.

Lattice search algorithm: the typical representatives are TANE [25], FUN [26], and FD_Mine [27] algorithms. The search space is modeled as the lattice of attribute combinations to represent all candidate functional dependencies, and a bottom-up search strategy is adopted to verify the candidate functional dependencies at each layer. The time complexity of the lattice search algorithm mainly depends on the size of the lattice, and the size of the lattice depends on the number of attributes of the dataset. Therefore, the lattice search algorithm has better row scalability and is suitable for large-scale datasets with fewer columns.

Difference set and consistent set algorithm: the typical representatives are Dep-Miner [28] and FastFDs [29] algorithms. Based on the comparison between tuples, the consistent set and the difference set are obtained, and finally the candidate functional dependency is verified according to the difference set. The time complexity of difference and consistent set algorithms depends on the number of tuples. Therefore, the difference set and consistent set algorithms have better column scalability and are suitable for small-scale datasets with many columns.

Hybrid Algorithm: HyFD [30] uses a hybrid discovery strategy to combine the advantages of the lattice search algorithm and the difference set and consistent set algorithms, and has better scalability in rows and columns. HyFD first generates a consensus set from the sampled data, identifies candidate functional dependencies from the consensus set, and uses FDTree to represent the corresponding attribute set. Then, HyFD is transformed into the lattice search algorithm, and candidate functional dependencies are verified by traversing the FDTree.

Approximate functional dependency discovery. In 1992, Kivinen and Mannila [31] first proposed an error metric for approximate functional dependencies. Subsequently, CORDS [32] automatically discovered unary approximate functional dependencies from relational data. To further speed up the discovery of approximate functional dependencies, the authors of [33] used heuristics to prune the candidate space of approximate functional dependencies. Mandros and Boley [34] represented the approximation of functional dependencies more precisely by scores.

The authors of [35] use a machine learning approach to infer approximate functional dependencies by comparing tuples with each other. The method finds all conflicting functional dependencies by tuple pair comparison, applies an error threshold to remove infrequent conflicting tuple pairs, and finally, inferring approximate functional dependencies from the remaining conflicting tuple pairs.

In recent work, Caruccio and Deufemia [36] proposed a new candidate approximate functional dependency verification method to discover multiple types of approximate functional dependencies by constructing a difference matrix of attributes. AFDDPar [37] proposed a parallel approach in a distributed environment for discovering approximate functional dependencies in a distributed environment, balancing the load of individual nodes before data redistribution, and pruning candidate approximate functional dependencies quickly after data redistribution.

## 3. The Distributed Algorithm for Mining Functional Dependency

In this chapter, a description of the distributed functional dependency discovery problem and a general overview of the algorithm DisTFD are given. In this paper, functional dependency discovery is carried out in a distributed big data environment, a distributed processing method is designed, and intermediate results are reasonably stored. On the premise of ensuring the correct rate, the load of each computing node is balanced as much as possible to reduce the time consumption of the algorithm.

### 3.1. Algorithm Architecture Overview

**Figure 2:**
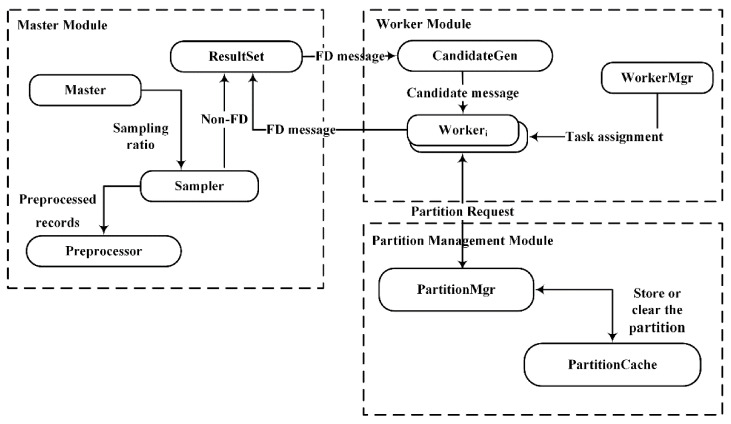
DisTFD logical structure diagram.

The algorithm DisTFD consists of multiple components, which are divided into different logical modules. The framework of the algorithm DisTFD is shown in Figure 2.

The components of DisTFD can be divided into three modules: Master Module, WorkerModule, and Partition Management Module.

The Master Module mainly performs data input, output, control sampling ratio, and data preprocessing. The master module can only be located on the master node. The worker module has several work nodes, which are mainly responsible for data storage and generation and verification of candidate function dependencies, and send the verification results to the master node. The partition management module merges the results calculated by multiple worker nodes, and stores the partition used for verifying candidate functional dependencies.

The specific functions of the components in the three modules are as follows:

**ResultSet.** The ResultSet component stores the invalid functional dependencies and the valid functional dependencies as two sets, respectively.

**CandidateGen.** The CandidateGen component generates candidate functional dependencies in the form of the lattice and sends the candidate functional dependencies to each worker node. After each validation, CandidateGen prunes candidate function dependencies according to the validation results in the ResultSet.

**Sampler.** The Sampler component samples the data according to the ratio set in the master node and is responsible for verifying the received candidate FD on the sampling data set $D^{\prime }$. If the verification result is true, the candidate function will be sent to the work nodes for further verification. If the verification result is false, the candidate functional dependency will be sent to the ResultSet.

**PartitionMgr.** The PartitionMgr component accepts the request for partition by the work node, and if there is a partition of the request in the PartitionCache, it will be sent to the corresponding work node. If the requested partition does not exist in PartitionCache, Worker will calculate the partition and merge calculation results by PartitionMgr. Then, PartitionMgr stores calculated partitions in the PartitionCache, and periodically clears the partitions that have not been accessed for a long time.

**Worker.** The Worker component verifies the candidate functional dependencies, and sends the result to the ResultSet and requests a new verification job from CandidateGen.

**WorkerMgr.** The WorkerMgr component records the load of each node after data redistribution. When the node load is unbalanced, the task assignment algorithm is called to assign the task to achieve load balance.

This paper proposes a distributed functional dependency discovery algorithm DisTFD based on attribute space traversal as shown in Algorithm 1:
**Algorithm 1**. Distributed Functional Dependency Discovery Algorithm DisTFD**Input:**$\;\mathrm{dataset}\;D=\left(D_{1},\ldots ,D_{n-1},D_{n}\right),\;\mathrm{attribute}\;\mathrm{set}\;X$ **Output:** Minimum non-trivial functional dependency set *Σ*      /* Set the sampling ratio *n* data preprocessing output sorted attribute set*/$D^{\prime }\;=\;\mathrm{Sampler}(D,\;n$)/* Data preprocessing output sorted attribute set */SortedAttribute = Pre_processing$\left(D^{\prime },X\right)$/* Generate candidate function dependencies */Candidate_FD = CandidateGen (*X*)SamplingValidate (φ, *D*′)**for** each $A_{i}\in SortedAttribute$ **do**        ReDistributedDataSet$(D,\;A_{i}$)        $\;\mathrm{if}\;(A_{i}\in SkewAttribute$){                Assignment (*D*)        }/* task assignment to balances the load of worker nodes*//*Verify that each function in the candidate space depends on φ∈Candidate_FD*/        **if** (GlobalValidate(φ, D) == true) {                Pruning(Candidate_FD, φ)                Σ=Σ∪φ        }**end for****Return***Σ*


### 3.2. Data Preprocessing

The preprocessor preprocesses the data, including statistical attribute cardinality and attribute value frequency. In the case of a large amount of data and distributed storage, it is necessary to summarize the results for all data statistics multiple times, which make the cost extremely high. Therefore, this article counts attribute-related information on the sampling data set and will introduce the sampling method in Section 3.3.

The number of types of attribute values is called the cardinality of the attribute, and the number of tuples corresponding to each attribute value is called the frequency of the attribute value. Based on the statistics of the cardinality and frequency information, the skewness of each attribute is then calculated. Given an attribute A, let c be the cardinality of attribute A, V be the set of all values of attribute A, $frequency\left(V_{k}\right)$ represents the frequency of the k-th value of attribute A, then the skewness of attribute A is expressed as:

(1) $$Inc\left(A\right)=\underset{1\leq k\leq c}{max}\frac{frequency\left(V_{k}\right)}{n}\;$$

where,$k\in \left[1,c\right]$, n is the total number of tuples in the dataset. The data preprocessing process is shown in Algorithm 2.
**Algorithm 2**. Pre_processing**Input:**$\mathrm{sample}\;\mathrm{data}\;\mathrm{set}\;D^{\prime },\;\mathrm{attribute}\;\mathrm{set}\;X$ **Output:** Sorted attribute set SortedAttribute Set the Skew threshold t**For**$A_{i}\in X$ **do**        $Inc\left(A_{i}\right)=\underset{1\leq k\leq c}{max}\frac{frequency\left(V_{k}\right)}{n}$       $\;\mathrm{If}\;(Inc\left(A_{i}\right)>t)\{\;SkewAttribute\leftarrow \;A_{i}$}     **else{** $NonSkewAttribute\leftarrow A_{i}$}**end for**SortByCardinality(NonSkewAttribute)SortedAttribute←NonSkewAttribute∪SkewAttribute**Return** SortedAttribute


After calculating the skewness of each attribute, the attributes are divided into Skew attribute and non-Skew attribute according to the given threshold. Then, sort all the attributes, and specify that the Skew attribute is ranked after the non-Skew attribute.

### 3.3. Sampling Validation Framework

Sampling refers to taking a part of the population of the research objects for investigation or statistics according to a certain procedure, so as to make inferences about the population of the research objects. In this paper, the statistical attribute information of the sampling data set reflects the situation of the attribute in the overall data set.

Sampler uses systematic sampling [38] to sample population data. According to the preset sample size n, determine an integer k closest to N/n, randomly select an integer r in the range of $\left[1,k\right]$ as the starting unit of the sample, and then select a unit every k as a sample unit until n samples are drawn.

The size of the sampled data set $D^{\prime }$ is much smaller than the overall data set D and is only stored on the master node. Therefore, the cost of functional dependency discovery on the sampled data set $D^{\prime }$ is small. The functional dependencies found in D and $D^{\prime }$ have the following two properties:

1. Completeness: A functional dependency φ that holds on D also holds on $D^{\prime }$.
2. Minimality: The minimum functional dependence φ that holds on $D^{\prime }$, if the functional dependence holds on D, then the functional dependence φ is also the smallest functional dependence on D.

According to the above two properties, the invalid or non-minimum functional dependencies can be quickly verified in the sampled data set, saving the time of distributed verification and improving the efficiency of the algorithm.

### 3.4. Search and Prune

**Figure 3:**
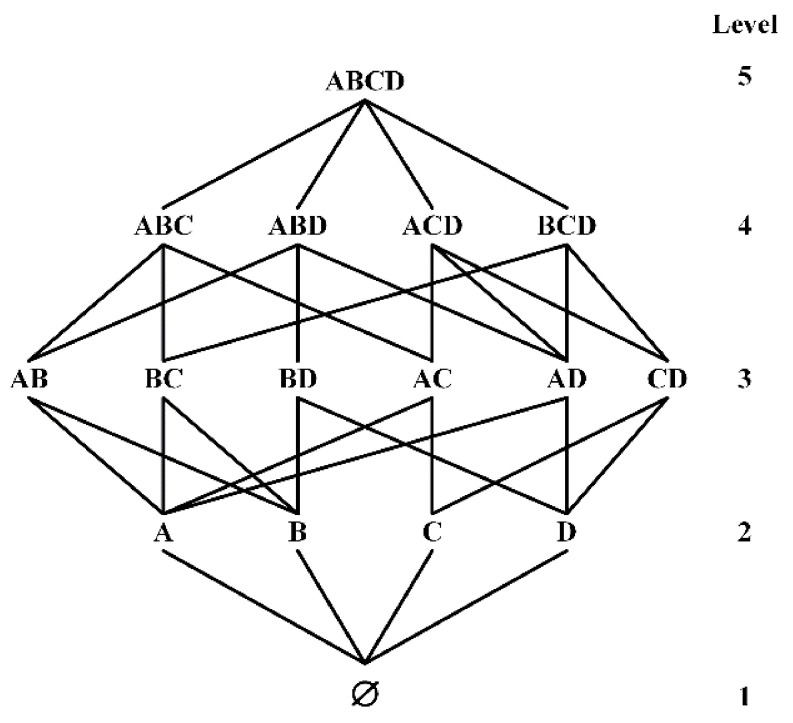
Candidate FDs composed of attribute sets A, B, C, D.

The row-efficient functional dependence discovery algorithm is appropriate for large-scale data sets with many tuples. Therefore, this paper uses the lattice of TANE, FUN and other algorithms to generate candidate functional dependence search space. Given a relational schema $R=\left\{A,B,C,D\right\}$, all of its candidate functional dependencies are shown in Figure 3.

The LHS of the candidate FDs is all possible attribute combinations in R, the connection between the first node of Level-5 and the first node of Level-4 represents the candidate function dependency ABC→D, the connection between the first node at level 3 and the first node at level 2 represents the candidate function dependency A→B, and so on.

**Lemma** **1.** *Given the attribute set $\left\{A_{1},\ldots ,A_{n}\right\}$ defined on the relational schema R, then the number of all non-trivial minimum functional dependencies is $n*2^{n-1}-n$.*

**Proof** **of** **Lemma** **1:** Consider only the nontrivial minimal functional dependencies for which RHS has one property. For all candidate functional dependencies on the relation R, the number of attributes of the LHS takes the value $\left[1,n-1\right]$. The number of candidate functional dependencies of LHS with only one attribute is $C_{n}^{1}*C_{n-1}^{1}$, the number of candidate functional dependencies of LHS with two attributes is $C_{n}^{2}*C_{n-2}^{1}$, and the number of candidate functional dependencies of LHS with three attributes is $C_{n}^{3}*C_{n-3}^{1}$,…, and the number of candidate functional dependencies of LHS with n−1 attributes is $C_{n}^{n-1}*C_{1}^{1}$. Therefore, the total number of non-trivial minimum functional dependencies for which RHS has a property is:

$$\begin{array}{ll} C_{n}^{1}*C_{n-1}^{1} & +C_{n}^{2}*C_{n-2}^{1}+C_{n}^{3}*C_{n-3}^{1}+\ldots +C_{n}^{n-1}*C_{1}^{1}\\ & =1*C_{n}^{1}+2*C_{n}^{2}+3*C_{n}^{3}+\ldots +\left(n-1\right)*C_{n}^{n-1}\\ & =0*C_{n}^{0}+1*C_{n}^{1}+2*C_{n}^{2}+\ldots +\left(n-1\right)*C_{n}^{n-1}\\ & +n*C_{n}^{n}-n*C_{n}^{n}=\overset{n}{\underset{j=0}{\sum }}j*C_{n}^{j}-n*C_{n}^{n}\\ & =n*2^{n-1}-n \end{array}$$

□

When verifying candidate functional dependencies, most existing lattice searches verify candidate functional dependencies one by one in a bottom-up or top-down order and the set of candidate functional dependencies is pruned using the following lemma:

**Lemma** **2.** *Let X, Y, Z be the three attribute sets of the relation R. If Y⊂X and X↛Z, then Y↛Z.*

**Lemma** **3.** *Let X, Y, Z be the three attribute sets of the relation R. If Y⊂X and Y→Z hold, then X→Z holds.*

According to Lemma 2, the top-down search strategy can be used to prune functional dependencies that do not hold in lower levels. For example, it has been verified that functional dependencies ABC→D do not hold, then AB→D and AC→D do not hold. Therefore, if most of the functional dependencies at the upper level are valid and those at the lower level are not, then the top-down strategy will verify more useless functional dependencies and reduce the verification efficiency.

According to Lemma 3, the bottom-up search strategy can be used to prune the functional dependencies at higher levels. For example, it has been verified that the functional dependencies AB→D holds, then ABC→D must hold, and bottom-up search strategy can avoid the verification of non-minimal functional dependencies. However, when there are many lower levels functional dependencies that do not hold, the search space cannot be effectively pruned.

In this paper, we adopt the validation method in [39] and use a two-way alternating search validation strategy in the sampling validation process. The validation is alternated from both ends of the search space. It is assumed that there are n levels of candidate functional dependencies. DisTFD verify the Level-i (i≤n/2) firstly, if the verification result is true, Lemma 2 is used to prune the functional dependencies greater than the Level-i. Then, verify the Level-j (j=n+1−i), Lemma 3 is used to prune the functional dependencies smaller than the Level-j if the verification result is false, and then verify the Level-(i+1), and so on until all candidate functional dependencies are verified. For example, in the 4-attribute search space shown in Figure 3, the verification order is Level-2: ∅→A; Level-4: ABC→D; Level-2: ∅→B; …; Level-3: CD→B.

### 3.5. Global Validation

Candidate function dependencies verified by sampling are further verified using data redistribution.

#### 3.5.1. Partition Caching

Calculating the number of equivalence classes in a partition to verify candidate functional dependencies. For example, verifying X→Y requires comparing $\left|\Pi_{X}\right|=\left|\Pi_{XY}\right|$ for equality.

**Theorem** **1.** *A functional dependency*X→Y*hold if and only if*$\left|\Pi_{X}\right|=\left|\Pi_{XY}\right|$.

**Proof** **of** **Theorem** **1**: Since $\left|\Pi_{X}\right|=\left|\Pi_{XY}\right|$ by definition 4 and definition 5, the number of equivalence classes in X is equal to the number of equivalence class in XY so the total number of tuples contained in the X and XY equivalence classes is equal. That is, for any tuple $t_{i}$, if $t_{i}$ is in an equivalence class of X, then $t_{i}$ is also in the same equivalence class of XY, and $t_{i}\left[X\right]=t_{j}\left[X\right]$ is satisfied for two tuples if $t_{i}$ and $t_{j}$ in the same equivalence class $\left|\Pi_{X}\right|$, then $t_{i}\left[Y\right]=t_{j}\left[Y\right]$, in line with the definition of functional dependency, it can be concluded that X→Y is hold. □

The partition $\Pi_{XY}$ can be derived from $\Pi_{X}⋂\Pi_{Y}$, a process called computing the intersection of partition. As shown in Figure 1, $\Pi_{A}=\left\{\left\{1,3,4\right\},\left\{2,5\right\}\right\}$, $\Pi_{C}=\left\{\left\{1,4\right\},\left\{2,5\right\}\right\}$, the process of calculating $\Pi_{AC}$ is as follows: First, $\Pi_{C}$ is converted into the attribute vector $v_{c}=\left(1,2,0,1,2\right)$, the value that appears only once is coded as 0, and the other values are coded as 1, 2, …, n in sequence. Then, group the equivalence classes in $\Pi_{A}$ according to the value other than 0 in $v_{c}$, $\Pi_{A}=\left\{\left\{1,3,4\right\},\left\{2,5\right\}\right\}$ can be divided into $1\to \left\{1,4\right\}$ and $2\to \left\{2,5\right\}$. Finally, among all the obtained groups, groups with size greater than 1 form a new partition, $\Pi_{AC}=\left\{\left\{1,4\right\},\left\{2,5\right\}\right\}$. The computational complexity of this process is high and a large amount of intermediate data will be generated during the calculation process, resulting in a long calculation time. Therefore, this paper stores the intermediate results in the partition cache to avoid repeated calculations in the verification process.

**Figure 4:**
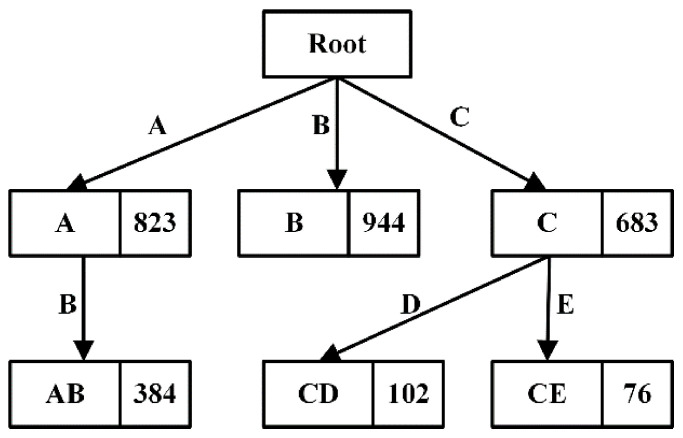
Prefix tree cache partition.

DisTFD stores the calculated partition in the prefix tree [40] shown in Figure 4 for easy query. Each node stores the partition corresponding to the path, and the number on the node indicates the size of the partition. In the above example, to calculate $\Pi_{AC}$, the attribute set $\left\{A,\;C\right\}$ is converted into an attribute list$\;\left(A,\;C\right)\;$according to the attribute order in the relational schema R, and then $\left(A,\;C\right)$ is used as a keyword to query in the prefix tree.

When using the partition in the cache, the following two rules should be followed:

1. When calculating the results, the number of partition intersections should be as few as possible.
2. In each calculation of $\Pi_{X}⋂\Pi_{Y}$, the $\left|\Pi_{X}\right|$ and $\left|\Pi_{Y}\right|$ should be minimized as much as possible.

Algorithm 3 gives the execution process of using partition to cache the calculation results under the two rules above.
**Algorithm 3.** RetrievePartition**Input:** Partition cache Cache, attribute set *X* **Output:** Partition $\Pi_{C}$Φ← Query the partition of all *X* subsets in the Cache$\Pi_{Y}\leftarrow$ Find the smallest stripped partitioning in *Φ*L←new list   C←Y**For**C⊂X **do**      $\Pi_{Z}\leftarrow$ Find the partition in *Φ* that satisfies $\left|Z\right|=max\left\{\left|X/C\right|\right\}$      $L=L.append\left(\Pi_{Z}\right)$   C←C∪Z**End for**Sort the partition in *L* in ascending orderC←Y**For**$\Pi_{Z}\in L$ **do**      $\Pi_{C\cup Z}\;=\Pi_{C}\cap \Pi_{Z}\;\;\;\;C\leftarrow C\cup Z$**Return**$\Pi_{C}$


In Algorithm 3, the partitions of all subsets of attribute set X are first queried in the cache and stored in the query result Φ, the smallest number of partitions is found as the starting unit of the partition intersection calculation. Next, according to rule 1, select the partition with the most newly added attributes in Φ to calculate the intersection, until all attributes in X appear at least once in the selected partition. Finally, the order of partition intersection calculation is determined according to Rule 2, the partition with a small number of equivalence classes should perform intersection calculation as soon as possible.

For example, assuming that $\Pi_{ABCDE}$ is currently calculated using the partition caching shown in Figure 4, $\Phi =\left\{\Pi_{A},\Pi_{B},\Pi_{C},\Pi_{D},\Pi_{E},\Pi_{AB},\Pi_{AD},\Pi_{CE}\right\}$ is searched in the prefix tree, and the smallest $\Pi_{CE}$ is selected as the starting unit, Then, select $\Pi_{AB}$ with the most newly added attributes, and then select $\Pi_{D}$. After the partition selection of the intersection calculation is completed, the order of intersection calculation is determined from small to large, and finally $\Pi_{ABCDE}=\Pi_{CE}\cap \Pi_{AB}\cap \Pi_{D}$ can be obtained.

When caching partitions, memory resources are usually limited. If all partitions are cached, excessive memory space may be occupied. Most partitions are only used for a period of time, DisTFD save the memory space by clearing partitions that are no longer used [41]. Each time the partition cache is returned, PartitionMgr records the access time of each partition and periodically clears the recently unused partitions.

#### 3.5.2. Task Assignment and Validation

Select the sorted attributes in turn as public attributes for data redistribution. In the process of data redistribution, the tuples with the same value on the common attribute are sent to the same node by calculating the hash value of the common attribute value. When the non-Skew attribute is used as the public attribute, it is directly verified after data redistribution, and when the skewed attribute is selected as the public attribute, DisTFD assignment the task based on the greedy strategy to achieve load balancing [42].

**Figure 5:**
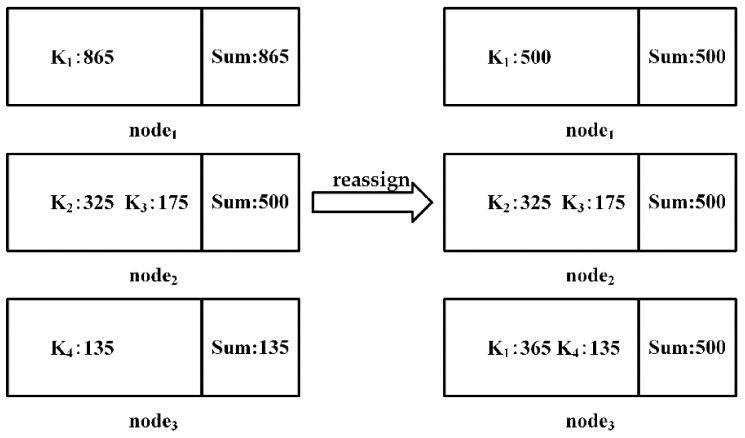
Task assignment to achieve load balancing.

Each attribute value of the public attribute is represented by $key_{i}\left(1\leq i\leq m\right)$, and the process of the task assignment shown in Figure 5 is as follows:

1. Sort keys from small to large according to the frequency of each attribute value counted in data preprocessing.
2. Add up all key frequencies to calculate mean Avg relative to the number of nodes.
3. Traverse the key, if the key frequency is greater than Avg, split it and assign it to a node with a load of 0, record the corresponding relationship between the key and the node allocation, and subtract the Avg from the frequency of the key. Repeat this step until the frequency of the key is less than Avg. If the frequency of the key is not 0, the key is re-inserted into the queue.
4. Repeat step 3 until all keys with a frequency greater than avg are processed
5. Select the remaining nodes that are not involved in step 3, traverse the key queue and find the sum of the node load and key frequency, if Sum is less than Avg, assign the key to the current node, and Sum is used as the load of the current node, then delete the information of the key in the queue. Repeat the above steps until all keys in the queue are processed.
6. Repeat step 5 to balance the load of the remaining nodes, and record the correspondence between keys and node assignments.

Algorithm 4 describes the process of Task assignment. Lines 1–3 calculate the sum of the key frequencies and calculate the average load Avg on m nodes. Lines 4–11 split the partitions with a load greater than the average, and record the assignment relationship between keys and nodes. Lines 12–20 traverse the Key queue, merge the partitions with a load less than the average, and record the relationship between keys and node assignment.
**Algorithm 4.** Assignment**Input:** dataset $D=\left(D_{1},\ldots ,D_{n-1},D_{n}\right)$
**Output**: true
Read the frequency of Key in preprocessing and record it to KfreqKlist = SortByFreq (Kfreq)Avg = Sum/m/* Split Keys with greater than average frequency*/**for** each Key∈Klist    **if**  (Key.size>Avg)      **For** Key.size>Avg**do**/*Key is assigned to the node with a load of 0*/        Node.add(Key, i) Key.size −=Avg      **end for**/* Re-insert the split Key into the queue */    Klist.Sort (Key)   **Else** break**end for****for** each node do   **for** each Key ∈ Klist      **If** (Avg ≥ node.size + Key.size)         node.add (Key, i)  node.size+= Key.size         Klist.remove (Key)    **end for****end for****Return true**

**Figure 6:**
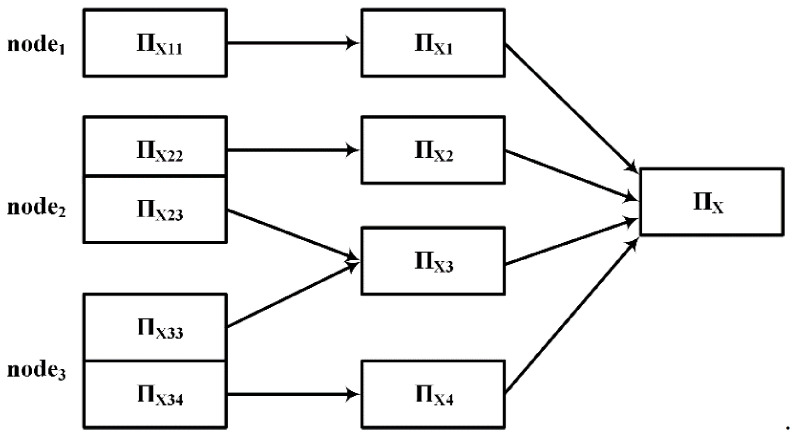
The process of merging the local equivalence classes of each node.

After the load balance is achieved, the local equivalence classes are obtained by computing the partitions in parallel at each node, and the local equivalence classes with the same value are merged. Finally, the partition of the candidate function dependent on LHS and LHS∪RHS is obtained. The process of merging local equivalence classes is shown in Figure 6.

Let A be a set of common attributes of candidate function dependencies, $d=\left(d_{1},\ldots ,d_{m-1},d_{m}\right)$ is the data after redistributing the attribute value of A, then

(2) $$\left|\Pi_{X}\right|=\sum_{j=1}^{m}\left|\Pi_{Xj}\right|$$

where, m is the number of keys,$\;j\in \left[1,\;m\right]$.

Algorithm 5 shows the process of parallel verification of selected function dependencies at each node.
**Algorithm 5.** GlobalValidate**Input:** dataset $D=\left(D_{1},\ldots ,D_{n}\right)$, candidate function dependency φ:X→Y **Output:** true or false
$\Pi_{Z}=RetrievePartition\left(X,Cache\right)$/* Each node computes $\Pi_{X/Z}$ in parallel*/Compute(d,X/Z)**for** each $\;\;\Pi_{X/Zij}$$\;\mathrm{in}\;node_{i}$    **If**$\;(\left|\Pi_{X/Zij}\right|$$\neq \left|\Pi_{X/Z\cup Yij}\right|$)        **Return** false    **for** $k\in \left[1,n\right]$        **If** (k≠i)           $\Pi_{X/Zj}\;\leftarrow \Pi_{X/Zij}\cup \Pi_{X/Zkj}$    **end for****end for**$\left|\Pi_{X/Z}\right|=\sum_{j=1}^{m}\left|\Pi_{X/Zj}\right|$**If**$\;(\left|\Pi_{X/Z}\right|$$\neq \left|\Pi_{X/Z\cup Y}\right|$)    **Return** false$\Pi_{X}\leftarrow \;\Pi_{X/Z}\cap \Pi_{Z}$$Cache\leftarrow \Pi_{X}$**If**$(\left|\Pi_{X}\right|=\left|\Pi_{X\cup Y}\right|$){    **Return** true}**else** {    **Return** false}

The input of Algorithm 5 is the redistributed data set D, the candidate function dependency φ:X→Y, and the output is the verification result. The algorithm first sends a request to the partitioned cache to obtain partial results, then computes the partition of the remaining attributes in X. Then, verify the candidate functional dependency on a single node, if the functional dependency is true on each node, merge the results with the same Key. Before storing the partition in the cache, the merged result is used to verify again to avoid storing invalid partition, and finally output the verification result of candidate function dependency.

## 4. Experiment

In this chapter, experiments are performed on real and synthetic datasets, and compared with other existing algorithms to verify the efficiency, scalability, and accuracy of the proposed algorithm.

### 4.1. Experimental Setup

In this experiment, a cluster consisting of 8 servers connected through a local area network is used. The configuration of each server is as follows: the CPU is Intel Xeon2 processor, 32GB memory, and the operating system is Ubuntu 10.4. The algorithm is written in Java and runs on Apache Spark and the HDFS distributed file system.

Three different types of datasets are used in the experiments: (1) A dataset with 0.5 million tuples generated by ONTS [43], the US Department of Transportation’s flight statistics. (2) Airline, a dataset with large number of columns, with 109 attributes and 0.5 million tuples [44]. (3) Synthetic dataset, a synthetic dataset Stud with 2 million tuples and 25 attributes. (4) Abalone, a small-scale dataset to evaluate the accuracy of the algorithm.

A summary of the experimental dataset is shown in Table 1.

### 4.2. Scalability

In this section, the scalability of DisTFD (Node scalability and Data scale scalability) is evaluated and compared with other algorithms.

**Figure 7:**
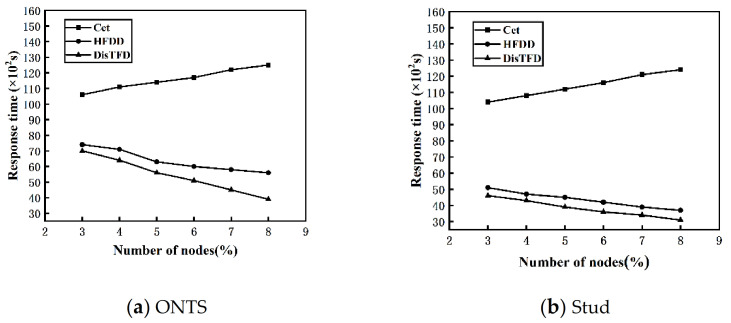
(**a**) Response time with different number of nodes (ONTS) (**b**) Response time with different number of nodes (Stud).

**Node Scalability.** By changing the number of nodes $\left|V\right|$, $3\leq \left|V\right|\leq 8$, the dataset scale is fixed, evaluate the scalability of this algorithm to the number of nodes. Figure 7a,b show the response times of algorithms Cet, HFDD and DisTFD under different numbers of nodes

As shown in Figure 7a,b, as the number of nodes increases, the response time of the algorithm HFDD and the algorithm DisTFD decreases significantly, and the response time of the algorithm Cet increases slowly. The algorithm Cet verifies the candidate functional dependencies by concentrating the data into master node. When the number of nodes increases, the data of each node migrates to the master node, the amount of data migration becomes larger and the load is unbalanced, which leads to an increase in response time. Algorithm HFDD and algorithm DisTFD verify candidate function dependencies in parallel, so as the number of nodes increases, the response time will be significantly reduced, but when the number of nodes is same, algorithm DisTFD is more efficient than algorithm HFDD. The results show that the algorithm DisTFD has better node scalability.

**Figure 8:**
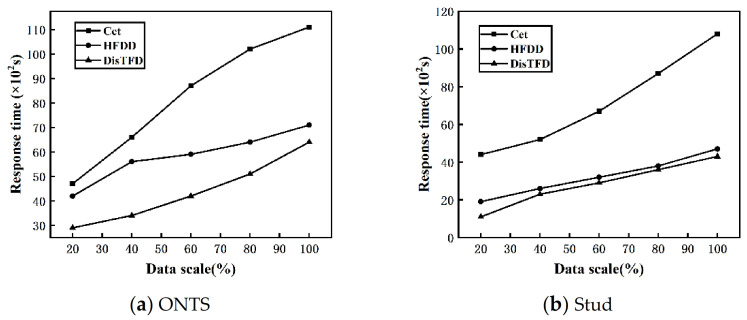
(**a**) Response time at different data scale (ONTS) (**b**) Response time at different data scale (Stud).

**Data scale scalability.** By changing the scale of the data set $\left|D\right|$, the scalability of the algorithm for the data scale is evaluated. The fixed number of nodes $\left|V\right|=4$, and the value range of the data scale is 20–100%. Figure 8a,b show the response times of algorithms Cet, HFDD and DisTFD under different data scales, respectively.

From Figure 8a,b, it can be concluded that with the expansion of the data scale, the response times of algorithms Cet, HFDD, and DisTFD show an increasing trend. Under the same conditions, the distributed discovery algorithms HFDD and DisTFD have less response time than the centralized discovery algorithm Cet. Compared with the algorithm HFDD, the algorithm DisTFD has a significant improvement in execution efficiency. From the above, it can be concluded that the algorithm DisTFD proposed in this paper has better scalability in terms of data scale.

### 4.3. Evaluation of Accuracy

In this section, we evaluate the accuracy of the algorithm by comparing the results of algorithms Cet, HFDD, and DisTFD with those of the TANE [25] algorithm, respectively, using the method in the literature [45].

We consider Precision, Recall, and F1measure as the metric of algorithm accuracy. The confusion matrix for classification results is shown in Table 2. Precision, Recall, and F1measure can be calculated as:

(3) $$Precision=TP/\left(TP+FP\right)$$

(4) $$Recall=TP/\left(TP+FN\right)$$

(5) $$F1measure=2*Precision*\;Recall/\left(Precision+Recall\right)$$

As shown in Table 2, the results can be divided into four categories: true positive (TP), false positive (FP), true negative (TN), and false negative (FN). We take the results of algorithm TANE as Truth, and the results of algorithms Cet, HFDD, and DisTFD as prediction values, and calculate Precision, Recall, and F1measure of the three algorithms and compare them, respectively. The comparison results are shown in Table 3.

As shown in Table 3, the algorithms Cet, HFDD, and DisTFD have little difference in Precision, Recall, and F1measure, and the F1measure of DisTFD is slightly improved, indicating that all the above algorithms have higher accuracy, but the algorithm DisTFD is more efficient with similar accuracy.

### 4.4. Evaluation of Performance

In this section, the effectiveness of the proposed method is evaluated by two sets of experiments, respectively.

**Figure 9:**
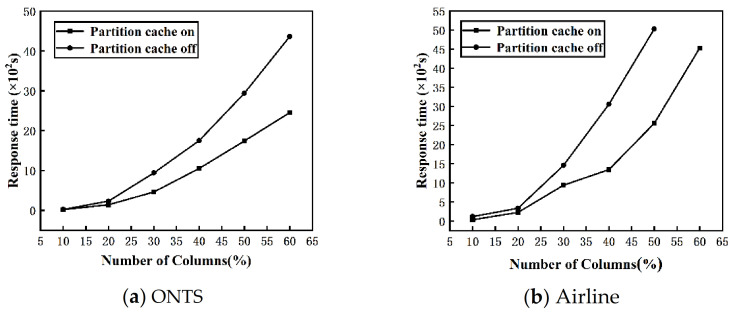
(**a**) Evaluation of effectiveness of partition cache (ONTS) (**b**) Evaluation of effectiveness of partition cache (Airline).

**Evaluation of partition cache.** By changing the number of columns in the ONTS and Airline datasets, we evaluate the effect of turning off and on the partition cache on the response time of the algorithm. The fixed number of nodes $\left|V\right|=4$, and the range of the number of data columns is 10–60%. Figure 9a,b shows the change of the response time of the DisTFD algorithm with the partition cache turning on or off as the number of columns increases.

As shown in Figure 9, when the number of columns is large, partition caching can significantly reduce the response time of the algorithm. When the number of columns is small, the response time of turning on or off the partition cache does not change significantly. As the number of columns increases, the partition cache significantly improves the execution efficiency of the algorithm.

**Figure 10:**
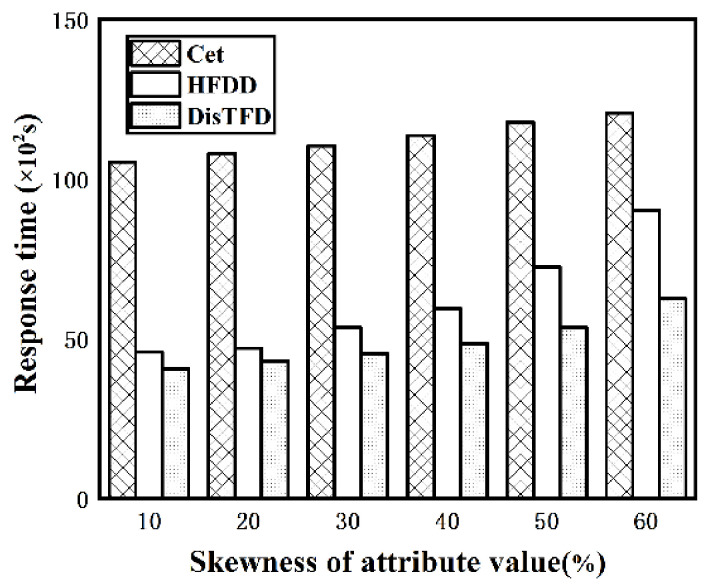
Evaluation of effectiveness of load balancing.

**Evaluation of load balancing.** By injecting attribute values with different skewness into the synthetic dataset Stud, the performance of the algorithm under different uniformity of attribute values is evaluated. The fixed number of nodes $\left|V\right|=4$, and according to the ratio of the number of tuples corresponding to the attribute value with the largest attribute value at the left end of the functional dependence to the total number of tuples in the data set from the lowest 10% to the highest 60%, the experiment is carried out. Figure 10 shows the response time of algorithms Cet, HFDD and DisTFD under the different skewness of attribute values.

As shown in Figure 10, the response time of algorithm Cet increases slightly with the increase in skewness, and the response time of algorithm HFDD increases significantly in the case of larger skewness. However, the algorithm DisTFD has no significant change in response time as the skewness increases. Therefore, the algorithm DisTFD has better performance in the case of uneven distribution of attribute values.

## 5. Conclusions and Future Work

Aiming at the problems existing in the process of centralized functional dependency discovery, this paper proposes an algorithm to discover functional dependencies from distributed data. This paper proposes a functional dependency discovery algorithm suitable for distributed data, focusing on reducing the response time of distributed functional dependency discovery. In order to improve the efficiency of functional dependency discovery in a distributed environment, the intermediate results in the calculation process are stored in the cache to reduce the repeated calculation of equivalence classes. Balance the load during the verification process to avoid inefficiencies caused by the unbalanced load. The proposed algorithm is validated on real and synthetic datasets. The results show that the algorithm has good scalability in terms of node and data scale, and significantly improves the execution efficiency compared with existing methods. In future work, we will consider discover approximately functional dependencies and discover functional dependencies in the case of incomplete data. In addition, how to improve the column scalability of the algorithm is also a problem that needs to be considered.

## Figures and Tables

**Table 1:** Summary of experimental dataset.

DataSet	#Tuples	#Attributes
ONTS	0.5	64
Airline	0.5	109
Stud	2	25
Abalone	0.004177	9

**Table 2:** Confusion matrix for classification results.

Truth	Predicted	
	Positive	Negative
Positive	TP	FN
Negative	FP	TN

**Table 3:** Comparison of Cet, HFDD, and DisTFD accuracy on Abalone.

	Precision	Recall	F1measure
Cet	0.9852	0.9708	0.9780
HFDD	1	0.9781	0.9890
DisTFD	1	0.9854	0.9926
